# Chronic Idiopathic Axonal Polyneuropathy: Current Evidence and Future Directions

**DOI:** 10.3390/cells15201911

**Published:** 2026-10-20

**Authors:** Bogdan Bjelica, Stojan Peric, Ivo Bozovic, Sonja Koerner, Milica Vukojevic, Susanne Petri, Aiden Haghikia

**Affiliations:** 1 Department of Neurology, Hannover Medical School, 1, Carl-Neuberg-Strasse, 30625 Hannover, Germany; 2 PRACTIS Clinician Scientist Program, Dean’s Office for Academic Career Development, Hannover Medical School, 30625 Hannover, Germany; 3 University Clinical Center of Serbia-Neurology Clinic, 11000 Belgrade, Serbia

**Keywords:** chronic idiopathic axonal polyneuropathy, idiopathic peripheral neuropathy, axonal polyneuropathy, diagnosis, pathophysiology, phenotyping

## Abstract

Chronic idiopathic axonal polyneuropathy (CIAP) is one of the most common diagnostic categories encountered in patients with chronic polyneuropathy, yet it remains insufficiently defined and incompletely understood. CIAP has been regarded as a slowly progressive, length-dependent, predominantly sensory or sensorimotor axonal polyneuropathy affecting middle-aged and older adults after exclusion of recognized acquired and hereditary causes. Increasing evidence suggests that CIAP is unlikely to represent a single disease entity. Instead, it may constitute a heterogeneous clinical phenotype arising from the convergence of several biological pathways on distal axonal degeneration. We summarize current knowledge regarding the definition, epidemiology, clinical presentation, diagnostic approach, pathophysiological hypotheses, and treatment of CIAP. Particular attention is given to metabolic and vascular risk factors, endoneurial microvascular alterations, neurotoxic 1-deoxysphingolipids, inflammatory and immune-mediated mechanisms, oxidative stress, genetic susceptibility, and age-related axonal vulnerability. We also discuss how deeper phenotyping and genetic testing may reclassify a subset of patients initially labeled as having CIAP. No disease-modifying therapy has been established for CIAP. Management focuses on symptomatic treatment and rehabilitation; whether risk-factor modification alters the disease course remains unproven. Future studies should aim to harmonize diagnostic criteria, improve biomarker-based phenotyping, and identify mechanism-defined subgroups that may become accessible to targeted therapeutic strategies.

## 1. Introduction

Peripheral nervous system disorders represent a broad and heterogeneous group of conditions involving primary dysfunction of anterior horn cells, peripheral nerves and neuromuscular junctions. Among these, polyneuropathies are the most prevalent, particularly in adults and older individuals, with an estimated prevalence of 5–8% [1]. Peripheral neuropathies are typically characterized by a progressive, length-dependent degeneration of peripheral nerves and may be either inherited or acquired. Virtually any condition that disrupts the neuronal microenvironment can impose stress on distal axons, which are especially vulnerable due to their long distance from the neuronal cell body and their dependence on an intact supply of nutrients and macromolecules [2]. While inherited neuropathies result from genetic defects, acquired peripheral neuropathies can result from a wide range of insults, including exposure to toxins, mechanical injury, or as a secondary consequence of other systemic disorders such as metabolic disorders, nutritional deficiencies, autoimmunity, or infections [3].

After excluding all known causes of peripheral nervous system damage commonly encountered in clinical practice, approximately one third of patients remain without an identifiable etiology. In other words, about every third patient with peripheral neuropathy leaves the clinic without a definitive etiological classification [4]. This subgroup of polyneuropathies has been described using various terms, including chronic polyneuropathy of undetermined cause, chronic idiopathic axonal polyneuropathy (CIAP), cryptogenic polyneuropathy, cryptogenic axonal polyneuropathy, unclassified peripheral neuropathy or even late-life chronic peripheral neuropathy of obscure nature [4,5]. In this review, CIAP refers to chronic idiopathic axonal polyneuropathy as defined in the following section. Studies using broader or related categories, such as idiopathic polyneuropathy or cryptogenic sensory polyneuropathy, are also considered where relevant, but their populations are not assumed to be equivalent to CIAP. When discussing these studies, we specify the population studied and distinguish their findings from evidence obtained in cohorts explicitly defined as CIAP.

Notably, none of the aforementioned terms refers to an “idiopathic demyelinating polyneuropathy.” This raises the question of how idiopathic axonal and idiopathic demyelinating polyneuropathies differ. Acquired demyelinating polyneuropathies are generally considered to be immune-mediated disorders, exemplified by chronic inflammatory demyelinating polyneuropathy (CIDP) [6], whereas the underlying pathophysiological mechanisms of acquired axonal polyneuropathies, particularly CIAP, remain largely unknown.

This review aims to provide an up-to-date overview of the current knowledge regarding CIAP, with a focus on clinical characteristics, diagnostic challenges, and emerging pathophysiological concepts.

## 2. Methods

This article was designed as a narrative review of CIAP. A literature search was performed in PubMed/MEDLINE from database inception to April 2026 using the search terms “chronic idiopathic axonal polyneuropathy”, “idiopathic polyneuropathy”, “cryptogenic polyneuropathy”, “cryptogenic sensory polyneuropathy”, and “idiopathic distal sensory polyneuropathy”. Additional targeted searches were performed in PubMed/MEDLINE for topics identified as relevant during manuscript preparation. Reference lists of key reviews and included articles were also manually checked for additional relevant publications. For the clinical evidence, articles were included if they were published in English, involved human subjects, and were clinically or biologically relevant to CIAP or closely related idiopathic chronic axonal neuropathy phenotypes. Selected experimental studies were additionally considered to provide biological context; their findings were distinguished from direct evidence in CIAP. Original studies, population-based studies, cohort and case–control studies, genetic studies, pathological studies, clinical trials, systematic reviews, consensus statements, and major narrative reviews were considered. Since this was a narrative review, no formal risk-of-bias assessment or meta-analysis was performed. The lead author and coauthors selected the final articles based on their relevance to CIAP and the objectives of this narrative review. The numbers of records retrieved and articles selected were not recorded. The single-database search may have missed relevant studies, and relevance-based article selection may have introduced selection bias. The final bibliography contains 135 references.

Evidence is synthesized qualitatively, with attention to study design, sample size, diagnostic criteria, clinical phenotype, comparator groups, and consistency across studies. Findings from cohorts explicitly defined as CIAP are considered more directly applicable to CIAP than findings from broader or related neuropathy populations. Conflicting results are discussed in the context of differences in populations and methods, while acknowledging when discrepancies remain unresolved. Associations and findings from small observational or pathological studies are not interpreted as establishing causal mechanisms.

## 3. Definitions and Diagnostic Approach

The term CIAP was first introduced in the early 1990s by Notermans et al. in a five-year follow-up study involving 75 patients, replacing earlier labels such as “late-life chronic peripheral neuropathy of obscure nature”. It is defined as a chronic axonal polyneuropathy that primarily affects middle-aged and older adults, presenting with sensory or sensorimotor symptoms and signs in the absence of an identifiable underlying cause, even after comprehensive diagnostic evaluation and prolonged follow-up [7]. CIAP predominantly affects large nerve fibers, with or without additional small-fiber involvement, and typically follows a slowly progressive course that does not usually result in marked disability and loss of ambulation [8].

There are currently no formal international guidelines for the diagnosis of CIAP, and most published studies have relied on diagnostic criteria proposed by Notermans and subsequent Dutch cohort studies [7,9]. The latest Cochrane review on drug therapy for CIAP used the following criteria: (1) Distal sensory or sensory and motor symptoms and signs of the limbs compatible with polyneuropathy; (2) Absence of systemic or neurological disease that could explain the symptoms or signs; (3) Chronic clinical course, not reaching a nadir in less than two months; (4) Exclusion of any recognized cause of the polyneuropathy, such as diabetes mellitus, renal insufficiency, biliary cirrhosis, alcohol abuse, medication use, toxic substance exposure, thyroid disorder, vitamin deficiency, malignancy, polycythemia, monoclonal protein, systemic autoimmune disease, inflammatory bowel disease, metabolic storage disease, sarcoidosis and amyloidosis, by medical history taking and clinical or laboratory investigations; (5) No indication of hereditary polyneuropathy; (6) Electrophysiological studies in agreement with axonal polyneuropathy without demyelinating features [10].

In 2020, the ACTTION diagnostic criteria were published to standardize terminology for research in idiopathic distal sensory polyneuropathy (iDSP) [11]. iDSP is an umbrella term for three subtypes: idiopathic mixed fiber sensory neuropathy (iMFN), idiopathic small-fiber sensory neuropathy (iSFN), and idiopathic large fiber sensory neuropathy (iLFN). CIAP and the ACTTION categories overlap but are not equivalent. The ACTTION framework concerns sensory neuropathies and generally excludes clinical muscle weakness, whereas CIAP may include mild motor involvement. The ACTTION criteria allow study-specific exceptions for motor involvement when appropriate to the research question and explicitly stated in the eligibility criteria. In iMFN, “mixed fiber” refers to combined small- and large-fiber sensory features, rather than mixed sensory and motor involvement. Under the ACTTION criteria, skin biopsy is not required to confirm iMFN when sensory nerve conduction studies are abnormal and the remaining clinical and exclusion criteria are fulfilled [11].

### 3.1. Clinical Approach

The optimal extent of laboratory testing in the diagnostic evaluation of chronic polyneuropathy remains uncertain. The EXPRESS study protocol describes a prospective multicenter observational study comparing the two diagnostic strategies, usual care, as determined by each patient’s neurologist, and a more limited workup proposed by a panel of neuromuscular experts. The study is designed to assess diagnostic concordance between the patient’s neurologist diagnosis and the panel diagnosis, as well as the costs and effects on management of a limited diagnostic workup. However, as only the study protocol has been published to date, the EXPRESS study has not yet provided evidence to establish or validate a minimal laboratory testing panel [12].

Commonly recommended initial laboratory investigations include a complete blood count, assessment of glycemia (fasting glucose and/or HbA1c), renal and liver function tests, thyroid-stimulating hormone, vitamin B12, inflammatory markers (erythrocyte sedimentation rate or C-reactive protein), and screening for monoclonal proteins with serum immunofixation. Recommendations vary between national guidelines and are supported by limited evidence. Further laboratory investigations should be guided by the clinical presentation and suspected underlying causes [13,14].

**Figure 1:**
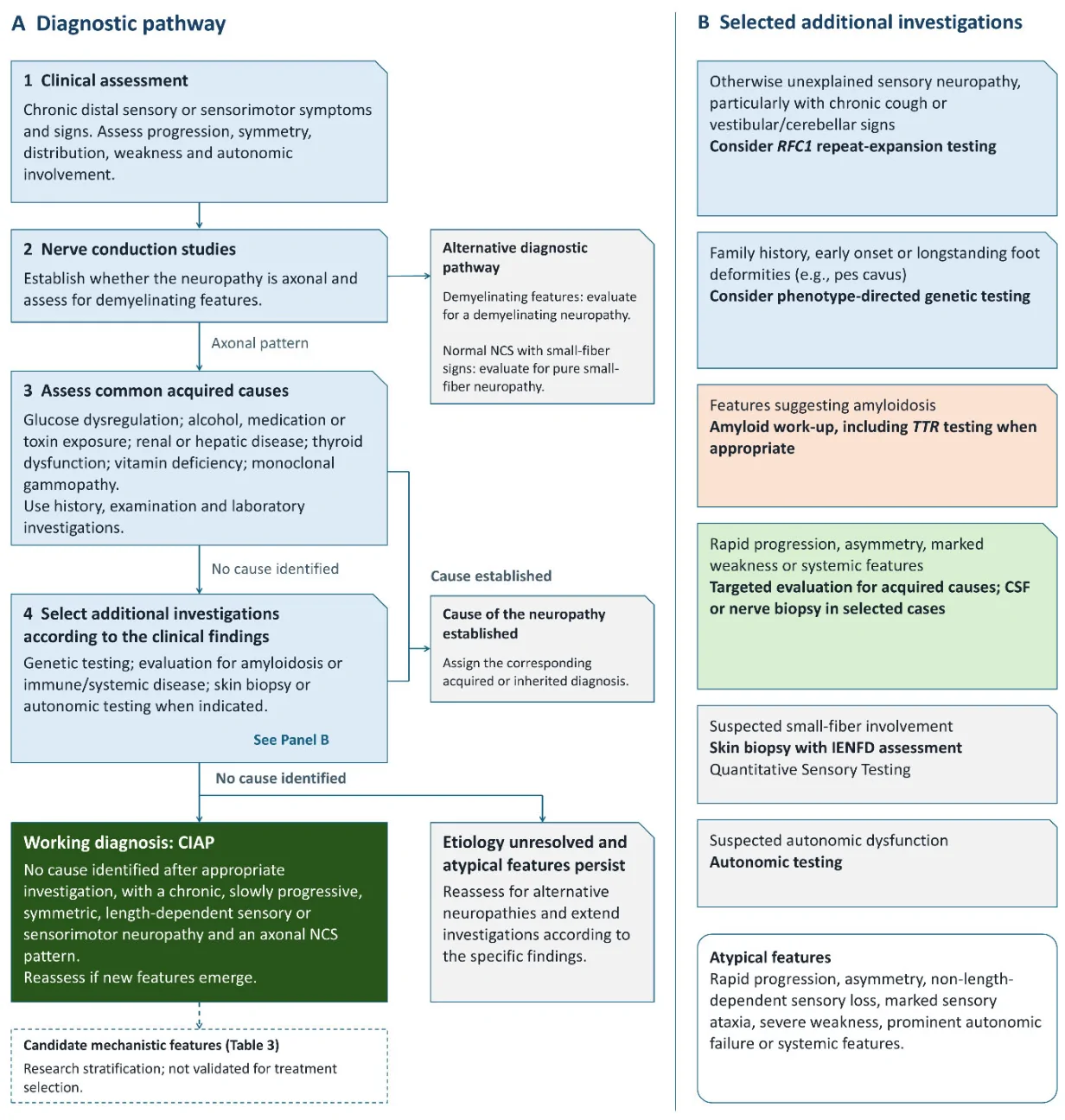
Proposed diagnostic approach to chronic idiopathic axonal polyneuropathy. Additional investigations are selected according to the clinical presentation and are not required in every patient. An abnormal screening result requires clinical interpretation before it is considered the cause of neuropathy. The dashed link to Table 3 indicates potential research phenotyping; these candidate features require validation for clinical classification and treatment selection. CIAP, chronic idiopathic axonal polyneuropathy; NCS, nerve conduction studies; IENFD, intraepidermal nerve fiber density; CSF, cerebrospinal fluid; RFC1, replication factor C subunit 1; TTR, transthyretin.

Given the lack of universally accepted diagnostic criteria and the heterogeneous terminology used in previous studies, a pragmatic stepwise approach is helpful in clinical practice. Electrophysiological studies should confirm an axonal pattern and exclude acquired demyelinating features, while common and treatable causes of polyneuropathy must be systematically assessed. In parallel, clinicians should actively search for red flags suggesting an alternative diagnosis, including rapid progression, asymmetry, non-length-dependent involvement, prominent autonomic dysfunction, marked sensory ataxia, severe weakness, systemic features, family history, or associated chronic cough, vestibular, or cerebellar signs. In such cases, targeted investigations, including genetic testing, amyloid work-up, skin biopsy, autonomic testing, cerebrospinal fluid analysis, autoimmune or paraneoplastic studies, or rarely, nerve biopsy, may be required. This proposed diagnostic workflow is summarized in Figure 1.

Hereditary transthyretin amyloidosis (ATTRv) should be considered in otherwise unexplained progressive axonal polyneuropathy. *TTR* gene sequencing is particularly relevant when neuropathy is accompanied by autonomic dysfunction, unexplained weight loss or gastrointestinal symptoms, bilateral carpal tunnel syndrome, cardiomyopathy or cardiac conduction abnormalities, or a family history of amyloidosis. An absent family history does not exclude ATTRv amyloidosis. Genetic findings require interpretation alongside the clinical phenotype and assessment for amyloid deposition and systemic involvement. Timely recognition is important because disease-modifying treatments are available [15].

Detection of a monoclonal protein in a patient with axonal neuropathy does not by itself establish a causal relationship. IgM-mediated neuropathies typically show demyelinating features, whereas the association of IgG or IgA monoclonal gammopathy of undetermined significance (MGUS) with axonal neuropathy is often uncertain. Evaluation should therefore consider other causes of neuropathy and disorders associated with a monoclonal protein, particularly immunoglobulin light-chain (AL) amyloidosis and, when clinically indicated, POEMS syndrome (polyneuropathy, organomegaly, endocrinopathy, monoclonal protein and skin changes). The immunoglobulin isotype, electrophysiological pattern and systemic findings should guide further assessment, ideally in collaboration with hematology [16].

After exclusion of common acquired causes, additional investigations can be selected according to the remaining diagnostic question. Longstanding foot deformities, such as pes cavus or claw toes, may suggest a hereditary neuropathy. Their significance should be interpreted alongside age at onset, family history and the clinical and electrophysiological phenotype when considering genetic testing [14]. RFC1 repeat-expansion testing may be informative in otherwise unexplained sensory neuropathy, particularly with chronic cough or vestibular or cerebellar signs [17]. When small-fiber involvement is suspected, distal-leg skin biopsy with quantification of intraepidermal nerve fiber density (IENFD), interpreted against age- and sex-adjusted normative values, can provide structural evidence of small-fiber loss [18]. Thermal quantitative sensory testing (QST) provides complementary functional information but depends on patient responses and should be interpreted alongside clinical findings [2]. Autonomic testing, including quantitative sudomotor axon reflex testing (QSART), may help characterize suspected autonomic small-fiber dysfunction [2]. If no cause is identified and the clinical and electrophysiological findings remain compatible, CIAP can be retained as a working diagnosis, with reassessment if new features emerge. Unresolved atypical findings warrant further assessment. The candidate features in Table 3 may inform further research phenotyping, but their use for mechanistic classification or treatment selection requires validation.

### 3.2. CIAP, Pure Small-Fiber Polyneuropathy, and Subacute Sensory Neuronopathy: Similarities and Differences

CIAP, pure small-fiber neuropathy, and subacute sensory neuronopathy (SSN, or sensory ganglionopathy) are frequently encountered in clinical neurology as “unexplained” sensory disorders. Although they differ substantially with respect to their primary anatomical targets, temporal course, electrophysiological features, and associated conditions, they overlap clinically in several important aspects. All primarily affect the sensory nervous system, share a considerable “idiopathic” burden, and may represent different points along a spectrum of immune-mediated or degenerative disorders of the sensory peripheral nervous system [19]. Understanding the relationships among these entities is essential for accurate diagnosis, appropriate diagnostic work-up, and the design of meaningful clinical and translational research studies with the potential to identify therapeutic options. Clinical and electrophysiological patterns of pure small-fiber neuropathy, CIAP and SSN are summarized in Table 1.

## 4. Epidemiology

Contemporary epidemiological data on prevalence and incidence for the majority of polyneuropathies remain limited, and existing studies should be interpreted with caution, as many do not fully adhere to current diagnostic criteria. Studies from the Netherlands published in 2015 and 2016 provide incidence and prevalence data on polyneuropathy, including idiopathic cases. In one of these studies, Visser et al. reported an age-adjusted incidence rate of 31.6 per 100,000 person-years among individuals aged 40 years and older [23]. More than half of the CIAP patients were male, with a mean age of approximately 70 years. The authors noted a marked increase in the proportion of CIAP cases with advancing age compared to other polyneuropathies. They concluded that in routine neurological practice, approximately one in seven patients presenting with polyneuropathy at age 40–49 years has CIAP, increasing to nearly one in three among those aged 80 years or older. Hanewinckel et al. conducted a population-based study of 1310 community-dwelling middle-aged and elderly individuals residing in a defined district of Rotterdam, the Netherlands, using a standardized screening protocol that included a symptom questionnaire, a structured neurological examination, and brief non-invasive nerve conduction studies [24]. The authors reported a minimum prevalence of polyneuropathy of 4% in this population, with nearly half of the identified cases being classified as CIAP. Subsequent Rotterdam Study analyses provide further population-based evidence on chronic axonal polyneuropathy and peripheral nerve function, including findings in participants without polyneuropathy [25,26,27,28]. These results should not be interpreted as CIAP-specific incidence or prevalence estimates.

## 5. Risk Factors Associated with CIAP

### 5.1. Modifiable Risk Factors

#### 5.1.1. Cardiovascular Risk Factors

Various studies have identified associations between chronic axonal polyneuropathies and several vascular risk factors, including abnormal glucose metabolism (such as diabetes and prediabetes in diabetic/prediabetic polyneuropathy), abnormal lipid metabolism (hypercholesterolemia and hypertriglyceridemia), and metabolic syndrome. However, only a limited number of studies conducted before 2013 applied defined diagnostic criteria to specifically identify CIAP, making it difficult to determine whether these associations are directly applicable to CIAP.

As part of the ongoing Utrecht study of CIAP etiology, Visser et al. investigated the association with metabolic syndrome in 249 patients with CIAP and 709 controls. The authors reported that over half of the CIAP patients met the criteria for metabolic syndrome, compared to approximately one third in the control group. Among the components of metabolic syndrome, hypertension and abdominal obesity emerged as the most consistently associated factors [9]. Another Dutch study also found an association between CIAP and both vascular diseases (stroke, heart disease, peripheral arterial disease) as well as vascular risk factors (hypertension, hypercholesterolemia, current smoking) [29]. Furthermore, Hanewinckel et al. demonstrated in a population-based study that older age, elevated body weight, hypertension, and moderate kidney dysfunction may contribute to peripheral nerve dysfunction, even in individuals who have not yet developed clinical signs or symptoms of polyneuropathy [25]. In a subsequent study, the same group found that better vascular health, encompassing both modifiable health behaviors and health factors, was associated with a lower prevalence of chronic axonal polyneuropathy [26].

Regarding dyslipidemia, studies are somewhat contradictory. While some studies have reported an association between dyslipidemia and CIAP [29,30,31], others have not found such a link [32]. Earlier studies even suggested a possible connection between long-term statin use and idiopathic polyneuropathy, based on a population-based patient registry in which 166 cases of idiopathic polyneuropathy were identified [33]. However, more recent studies involving larger cohorts have not confirmed this association [34,35,36].

Diabetes mellitus is already established as the most common cause of chronic axonal polyneuropathy [37], and impaired glucose metabolism is frequently identified as the underlying cause in cases of polyneuropathy initially considered idiopathic [38]. Early studies in the 2000s suggested an association between idiopathic polyneuropathy and prediabetes or impaired glucose metabolism [39,40,41,42]. These studies included between 48 and 219 patients, with some not strictly adhering to the diagnostic criteria for CIAP, probably resulting in more heterogeneous cohorts. However, Visser et al. found no increased prevalence of impaired fasting glucose among CIAP patients compared to controls [9]. Moreover, the oral glucose tolerance test (OGTT) may have limited utility and reliability in identifying prediabetes and its association with idiopathic polyneuropathy, particularly when performed as a single assessment [43]. In prospective follow-up studies, no new diagnosis of diabetes mellitus was reported in cohorts initially comprising 75 patients with CIAP (mean follow-up, 4.7 years) [7] and 40 patients with chronic polyneuropathy of undetermined cause (follow-up, at least four years) [44].

#### 5.1.2. Nutrition, Vitamins and Alcohol Consumption

As a modifiable lifestyle factor, diet plays a crucial role in the development of chronic diseases like diabetes, vasculopathies, and disorders linked to vitamin deficiencies, or metabolic syndrome [27,45,46], all of which have been hypothesized to constitute risk factors for CIAP. Furthermore, nutritional neuropathies, i.e., those caused by hypovitaminosis (thiamine (B1), pyridoxine (B6), folate (B9), cobalamin (B12), and vitamin E), are routinely encountered in clinical practice [47]. Most of these, much like CIAP, present as length-dependent sensory axonopathies [47]. In a later nutrition analysis within the same Utrecht study, Visser et al. investigated nutrition as a potential risk factor for CIAP using a validated food frequency questionnaire that covered nutrient intake and alcohol consumption in 318 patients with CIAP and 636 matched controls. However, they found no evidence supporting this association [48]. Taams et al. further examined the relationship between diet quality (assessed using a validated 389-item food frequency questionnaire (FFQ)) and both the presence of chronic axonal polyneuropathy (99 patients) and peripheral nerve function, as measured by sensory nerve conduction studies in participants without definite polyneuropathy (1202 participants) [27]. Their findings confirmed the results of Visser et al., showing no association between diet quality and chronic axonal polyneuropathy. However, it remains unclear how many of the participants included in this study specifically met the criteria for CIAP.

Vitamin B6 (pyridoxine) is a water-soluble vitamin essential for amino acid metabolism and normal nerve function [49]. While vitamin B6 deficiency is an established cause of peripheral polyneuropathy [50], excessive intake has also been proposed as a potential contributor to CIAP following reports of distal sensory neuropathy in patients taking very high doses of vitamin B6 (100–6000 mg/day) [51,52]. However, subsequent studies do not support a clear association. In the vitamin B6 analysis within the Utrecht study, which included 381 patients with CIAP and 140 healthy controls, Visser et al. found more frequent use of vitamin B6-containing supplements among patients than controls, but no significant difference in vitamin B6 levels [53]. Similarly, Stewart et al. reported that moderately elevated plasma vitamin B6 levels were not associated with greater neuropathy severity in 261 patients with CIAP [54].

Alcohol-related peripheral neuropathy is thought to result mainly from the direct toxic effects of ethanol or its metabolites. It typically presents as a small-fiber neuropathy, although large-fiber involvement may occur, particularly in the context of malnutrition or vitamin B1 deficiency [55]. About half of individuals with chronic alcoholism are affected, but the threshold of alcohol exposure required to induce neuropathy remains unclear [55]. Current evidence suggests that moderate alcohol consumption is not a risk factor for CIAP [48], although individual susceptibility, including possible genetic predisposition, may influence the development of alcohol-related neuropathy.

#### 5.1.3. Chronic Obstructive Pulmonary Disease and CIAP

Chronic obstructive pulmonary disease (COPD) is increasingly recognized as a systemic rather than purely pulmonary disease [56,57]. Accordingly, peripheral nervous system involvement has been observed in patients with COPD [58,59,60]. However, a prospective controlled study included 345 patients with cryptogenic axonal polyneuropathy and concluded that their findings do not support the hypothesis that COPD is a risk factor for polyneuropathy [61].

### 5.2. Non-Modifiable Risk Factors

#### 5.2.1. Age

As outlined above, increasing age is a well-established, non-modifiable risk factor for chronic axonal polyneuropathy, including CIAP [23]. Both population-based and hospital-based studies from the Netherlands consistently demonstrate a steep increase in incidence and prevalence with advancing age, with CIAP predominantly affecting individuals aged ≥40–50 years [23,24]. Age at examination and age at symptom onset should therefore be considered robust, non-modifiable risk factors and key stratification variables in clinical and epidemiological CIAP research.

#### 5.2.2. Sex

Previous studies have reported a slight male predominance in CIAP [20,23]. However, there is no strong or consistent evidence that sex represents an independent, non-modifiable risk factor once common comorbidities such as diabetes, obesity, and other systemic conditions are accounted for. This contrasts with immune-mediated neuropathies, such as Guillain-Barré syndrome (GBS) and CIDP, in which male sex is consistently overrepresented [62].

#### 5.2.3. Height and Body Size

Among the anthropometric measures examined by Winsvold et al., height showed the strongest genetic correlation with idiopathic polyneuropathy [63]. The authors interpreted this finding as supportive of prior clinical observations indicating that taller individuals are more likely to develop symptomatic polyneuropathy for a given burden of risk factors, suggesting that increased axonal surface area may be a contributing mechanism [63,64]. While this hypothesis may also be relevant for CIAP, it has not yet been specifically investigated in pure CIAP cohorts.

#### 5.2.4. Race/Ethnicity

Reynolds et al. performed a cross-sectional secondary analysis including 8014 individuals (55% non-Hispanic White, 18% non-Hispanic Black, 24% Hispanic, 3% other). They found that non-Hispanic Black (odds ratio [OR] 1.39, 95% confidence interval [CI] 1.16–1.66) and Hispanic (OR 1.31, 95% CI 1.11–1.54) individuals had higher age- and sex-adjusted odds of polyneuropathy compared with White individuals. After further adjustment for metabolic, lifestyle, and social determinants of health, only Hispanic individuals maintained a significantly higher likelihood of polyneuropathy (OR 1.32, 95% CI 1.07–1.63) [65]. Similar data for CIAP patients are currently lacking.

Overall, associations with older age and selected cardiometabolic factors are supported by studies in CIAP, whereas evidence for several other proposed risk factors is inconsistent or derived from broader neuropathy populations. These observational findings do not establish causal relationships in CIAP.

## 6. Clinical Presentation of CIAP

The onset of CIAP is insidious, usually in the sixth decade of life, and the disease follows a slowly progressive course [21]. Patients usually do not develop severe disability [66].

### 6.1. Symptoms

Patients with CIAP typically present with predominantly sensory symptoms, with numbness being the most commonly reported complaint [21]. Due to involvement of large myelinated fibers, sensory ataxia and a subjective sense of tightness in the limbs may occur [21]. In addition, some studies have also demonstrated small-fiber involvement in CIAP [22], which can give rise to symptoms such as tingling, pins and needles, burning sensations, and neuropathic pain. Reported findings include abnormalities on thermal sensory and cardiovascular autonomic testing [22] and reduced distal-leg IENFD in a selected cohort with painful CIAP and suspected small-fiber involvement [67]. The symptoms are usually confined to, and first appear in, the distal lower extremities (length-dependent polyneuropathy), whereas symptom restriction to or onset in the upper extremities is very rare [68]. Pain is present in more than two thirds of patients and is most commonly described as a “nagging discomfort” [68]. In the study by Erdmann et al., which assessed pain in 91 patients with CIAP using two questionnaires, approximately two thirds of patients reported pain, with 43% experiencing neuropathic pain, 40% non-neuropathic pain, and 17% mixed pain [69]. Initial motor symptoms such as muscle cramps, stiffness, weakness, and wasting may also occur [8,21]. Autonomic test abnormalities and symptoms have been reported in small studies of CIAP [22,67], but these findings do not establish their frequency in unselected CIAP populations.

### 6.2. Signs

On neurological examination, patients with CIAP exhibit a fairly stereotyped pattern of distal, symmetric, predominantly large-fiber involvement, with variable small-fiber signs present in a subset of CIAP patients. Distal loss of vibration sense, typically at the toes and ankles, is often the earliest and most consistent finding, followed by abnormalities of light touch sensation [8,68]. Impaired position sense and a positive Romberg sign are somewhat less common but may also be observed [8]. The ankle tendon reflex is the most frequently absent deep tendon reflex; however, its absence is of limited diagnostic value in elderly individuals, in whom reduced or absent ankle reflexes may occur in the absence of polyneuropathy [8,68]. Generalized areflexia is less common in CIAP [8]. On motor examination, almost half of the patients demonstrate mild distal weakness, more frequently affecting the lower extremities. Among small-fiber-related symptoms, tingling or prickling sensations are most frequently reported [8,68], followed by pain and thermal dysesthesias, including burning or abnormal cold or warm sensations [8].

### 6.3. Electrophysiology

Nerve conduction studies (NCS) should not satisfy electrophysiologic criteria for demyelinating neuropathy [8,68,70]. Abnormal sensory NCS are encountered more commonly than altered motor NCS, with reduced sural sensory nerve action potential (SNAP) amplitudes being the most common sensory abnormality and reduced peroneal nerve amplitudes the most common motor NCS abnormalities. However, it should be noted that sural SNAPs are often absent in individuals over 80 years of age without any clinical symptoms of polyneuropathy, and thus nonrecordable sural responses in this age group are not necessarily proof of neuropathy [28].

Another important factor to consider when performing NCS in patients with CIAP is the temperature of the examined limb. Lower limb temperature leads to slower nerve conduction velocities, prolonged distal latencies, and increased amplitude and duration of potentials, particularly in sensory compared to motor nerves (SNAP > CMAP) [71,72]. Therefore, in patients with polyneuropathies, findings can easily be misinterpreted if limb temperature is not properly controlled. In patients with axonal polyneuropathies, cooling may reduce nerve conduction velocities into the range typically associated with demyelination and may increase predominantly sensory amplitudes to values within the normal range [71]. The use of temperature correction factors is common; however, these factors are primarily derived from studies in individuals with normal nerve function and may therefore not be applicable to diseased nerves. Hence, it is advisable to warm the limb rather than rely on a correction factor [71]. In a study of 19 patients with CIAP, Franssen et al. found that correction factors derived from healthy individuals overestimated nerve conduction velocity compared with measurements after warming [73]. A practical target is a skin temperature of at least 32 °C at the wrist and ankle before NCS [74]. If warming is required, one established method is immersion in circulating water maintained at 37 °C for 30 min, followed by repeat measurement and documentation of skin temperature before recording [70,74].

### 6.4. Natural History and Follow-Up

Available longitudinal studies suggest that CIAP usually progresses slowly, although functional limitations can increase. In the study by Notermans et al., with a mean follow-up of 4.7 years, clinical motor involvement developed in 7 of 28 patients (25%) who had a clinically sensory neuropathy at baseline. An alternative cause was identified in 4 of the original 75 patients (5.3%): hereditary motor and sensory neuropathy type 2 in two, chronic inflammatory demyelinating polyneuropathy in one, and alcohol-related neuropathy in one [7]. In another prospective cohort of 40 patients with chronic sensorimotor polyneuropathy of undetermined cause, no cause was identified during at least four years of follow-up. Thirty-four patients reported slight extension of numbness, seven reported slight worsening of distal weakness, and six remained unchanged; all remained ambulatory [44]. These estimates are cohort-specific and reflect the diagnostic investigations available at the time.

In a study comparing patients with different disease durations, Vrancken et al. reported that none of 127 patients with CIAP had a modified Rankin score of 4 or 5, including the 38 patients with a disease duration of at least 10 years; 35 patients used walking aids, including adapted footwear and orthoses [8]. In a prospective study of 92 CIAP patients, van Oeijen et al. found worsening in walking performance, daily step count and self-reported physical functioning over four years of follow-up. Sixty-five patients completed the four-year measurements; the 29.3% loss to follow-up should be considered when interpreting these findings [75]. Zis et al. studied 57 patients, including 49 with length-dependent neuropathy and eight with sensory ganglionopathy. In the length-dependent subgroup, the estimated mean annual declines were 3.2% for superficial radial and 4.7% for sural sensory nerve action potential amplitudes and 6.1% for tibial compound muscle action potential amplitudes [76].

## 7. Pathophysiology of CIAP—Current Understanding and Hypotheses

CIAP is unlikely to represent a single pathobiological entity. Rather, it may be regarded as a heterogeneous clinical phenotype that emerges when an aging peripheral nervous system with reduced axonal resilience is exposed to one or more additional “second hits”. Metabolic-vascular factors, dysregulated sphingolipid metabolism, inflammatory-immune mechanisms, and oxidative stress may contribute to a shared downstream process involving endoneurial microvascular dysfunction, chronic cellular stress, impaired axonal homeostasis, and ultimately progressive distal axonal degeneration.

**Figure 2:**
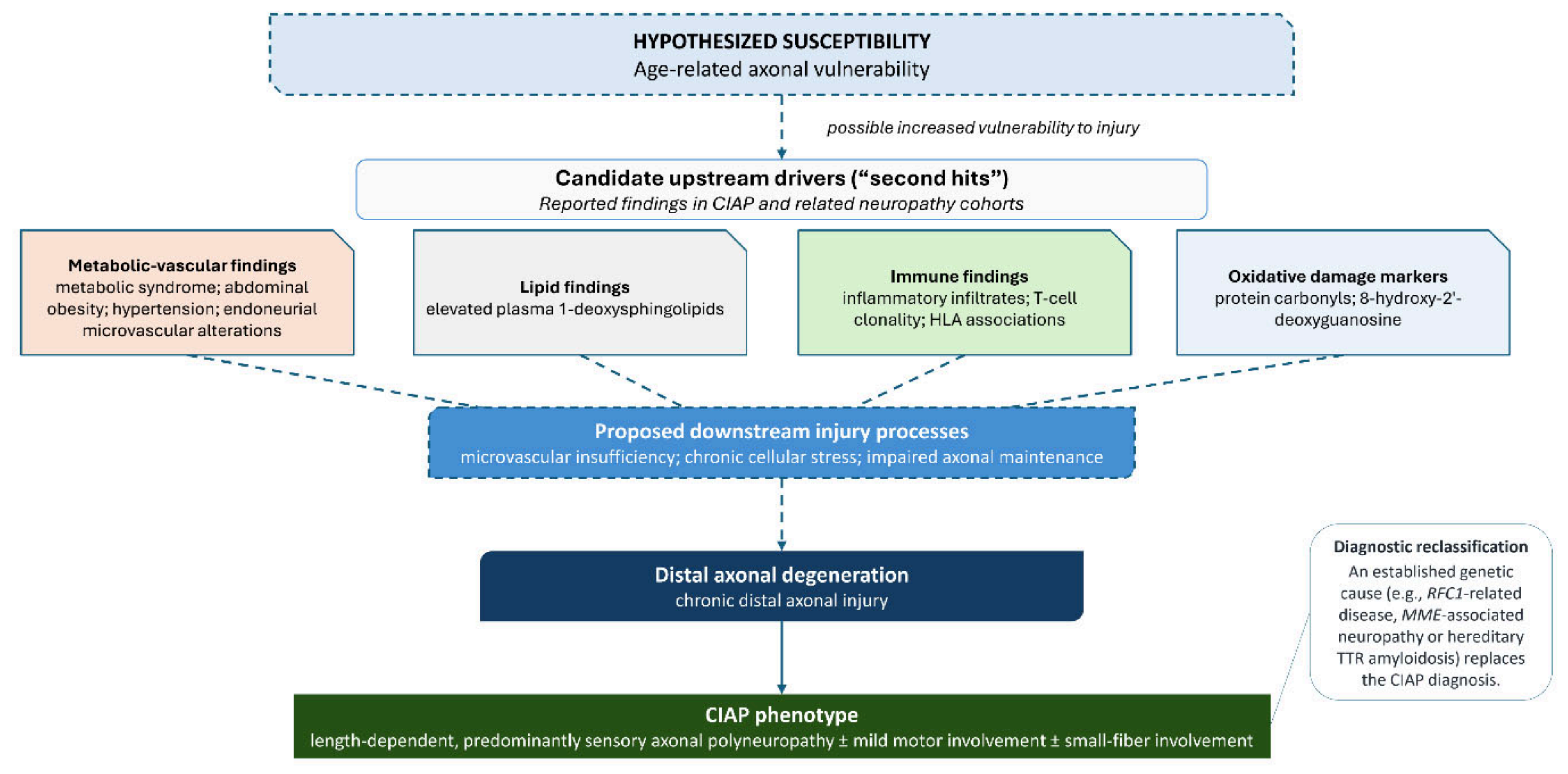
Hypothesized pathophysiological framework of chronic idiopathic axonal polyneuropathy. Solid outlines around the four finding domains denote reported observations in CIAP and related neuropathy cohorts; they do not grade the strength of evidence. The reported findings derive mainly from observational studies, including clinical association studies, biomarker comparisons and small pathological series. Dashed outlines identify hypothesized age-related axonal vulnerability and proposed downstream injury processes; dashed connectors indicate proposed mechanistic links. The evidence varies across pathways and populations and does not establish causality or validated mechanistic subgroups of CIAP. Study populations and limitations are summarized in Table 2. CIAP, chronic idiopathic axonal polyneuropathy; HLA, human leukocyte antigen; MME, membrane metalloendopeptidase; RFC1, replication factor C subunit 1; TTR, transthyretin.

This proposed multihit framework is summarized in Figure 2. It should be emphasized that the relationships shown are hypothetical and based predominantly on associative, biomarker, and small pathological studies; they do not establish causality, and several mechanisms may coexist within an individual patient. A proportion of patients initially labeled as having CIAP may subsequently receive a different diagnosis after deeper phenotyping or genetic testing, for example, *RFC1*-related disease, *MME*-associated neuropathy, transthyretin amyloidosis, or another late-onset inherited neuropathy. The following sections review the evidence relating to each proposed mechanism and its limitations, distinguishing findings from cohorts explicitly defined as CIAP from those obtained in broader or related neuropathy populations. Even among CIAP cohorts, differences in diagnostic criteria and exclusion procedures limit comparability. Findings from related populations are therefore considered potentially informative for CIAP but do not establish that the same mechanisms operate in CIAP. Selected studies, their populations, diagnostic criteria, principal findings, and limitations are summarized in Table 2.

Long peripheral axons depend on local ATP production and transport of organelles and other cargo over considerable distances. Experimental work illustrates how impaired metabolic support can compromise distal axonal maintenance. In mice, deletion of pyruvate kinase M2 in myelinating Schwann cells reduced lactate availability, impaired the increase in axonal mitochondrial ATP during activity, slowed mitochondrial transport and caused withdrawal of neuromuscular terminals despite preserved myelin [77]. These findings support a role for Schwann-cell metabolic support in axonal maintenance, but their relevance to CIAP remains to be established.

**Table 2:** Selected studies of risk factors and pathophysiology in CIAP and related neuropathy populations.

Study/Topic	Population and Sample	Diagnostic Definition	Principal Findings	Limitations and Applicability
Visser et al., 2013 [9] Metabolic factors	CIAP: 249 patients; 709 controls.	Distal, symmetric sensory or sensorimotor neuropathy for ≥6 months; no cause after extensive investigation; no hereditary clues; nerve conduction studies exclude demyelination.	Metabolic syndrome was more frequent in CIAP (55% versus 34%; age- and sex-adjusted OR 2.2, 95% CI 1.7–3.0). Hypertension and abdominal obesity were associated factors.	Evidence directly from a clinically defined CIAP cohort. Case–control associations do not establish that metabolic abnormalities cause CIAP.
Visser et al., 2017 [48] Nutrition	318 CIAP patients; 636 controls matched for age, sex and postal code. Sensory phenotype: 73%; sensorimotor: 27%.	CIAP criteria referenced to [9]: distal symmetric sensory or sensorimotor neuropathy for at least 6 months, no identified cause, and no demyelinating features on nerve conduction studies. Diabetes and average alcohol intake of at least 4 units/day were excluded.	A validated food-frequency questionnaire found no association between CIAP and the intake or density of the nutrients examined after adjustment for confounders and multiple testing.	Evidence from a defined CIAP cohort. Retrospective dietary recall and possible referral bias limit inference; the study does not test whether dietary changes alter CIAP progression.
Taams et al., 2019 [27] Diet quality	1650 Rotterdam Study participants: 99 with definite polyneuropathy and 1551 without definite polyneuropathy. Sural nerve analysis: 1202 participants without definite polyneuropathy.	Expert classification based on symptoms, neurological examination, nerve conduction studies and medical records. The comparison group combined no, possible and probable polyneuropathy. Known causes, including diabetes, were not excluded.	Overall diet quality was not associated with definite chronic axonal polyneuropathy (adjusted OR 0.99, 95% CI 0.88–1.12) or sural sensory nerve action potential amplitude.	Broader chronic axonal polyneuropathy population, not a CIAP-specific estimate. Questionnaire-based dietary assessment preceded neuropathy screening by a median of 5.1 years.
Hoffman-Snyder et al., 2006 [39] Glucose metabolism	100 consecutive patients with initially unexplained neuropathy; retrospective tertiary-clinic series. Comparison with published general-population prevalence estimates.	Sensory symptoms for more than 3 months; recognized causes initially excluded; nerve conduction studies excluded demyelination. The CIAP label included sensorimotor, sensory and small-fiber phenotypes, including possible small-fiber neuropathy with normal objective tests.	Oral glucose tolerance testing identified impaired glucose tolerance in 38 patients and diabetes in 24; abnormal fasting glucose was identified in 39 patients.	Broader definition than large-fiber CIAP, with subsequently identified diabetes and no concurrently recruited control group. Diagnostic yield does not establish that impaired glucose tolerance causes CIAP.
Hube et al., 2017 [31] Lipid metabolism and nerve pathology	30 CIAP; 28 diabetic or prediabetic neuropathy patients; 31 healthy controls. Biopsy subset: 10 CIAP and 7 diabetic/prediabetic neuropathies.	Symmetric, predominantly sensory distal neuropathy for >3 months; at most mild distal weakness; no demyelinating features or identified cause. Oral glucose tolerance testing excluded diabetes/prediabetes in CIAP.	Plasma 1-deoxysphingolipids were elevated versus healthy controls. Biopsy basal lamina thickness, CD68 and CD8 measures did not differ significantly between disease groups; leukocyte-common-antigen-positive cells were increased in CIAP.	CIAP label, but a shorter duration threshold than ≥6-month definitions. Small biopsy subset; no healthy biopsy controls. No comparison adjusted for metabolic syndrome was reported; independence from metabolic abnormalities and causal relevance remain uncertain.
Teunissen et al., 2000 [78] Microangiopathy	Sural biopsies: 18 CIAP; 4 diabetic neuropathy; 6 hereditary motor and sensory neuropathy type II (HMSN II); 10 autopsy controls.	Axonal degeneration with no cause after extensive clinical and laboratory evaluation. Detailed symptom duration and electrophysiology are not restated in this article.	Basal lamina area thickness was greater than in HMSN II (*p* = 0.01), but not significantly greater than in autopsy controls (*p* = 0.08). Thickness was similar to diabetic neuropathy and greater with peripheral arterial disease within CIAP.	Detailed CIAP eligibility criteria are not restated; small biopsy cohort; frequent vascular comorbidity. No association between basal lamina thickness and myelinated fiber loss. Observational vessel changes do not establish an ischemic cause of CIAP.
Samuelsson et al., 2016 [79] Microangiopathy	10 CIAP patients; 10 controls without sensory polyneuropathy; 11 inflammatory neuropathy controls (6 CIDP, 5 vasculitic neuropathy).	Electrophysiologically confirmed, predominantly distal axonal sensorimotor neuropathy; no cause after clinical and laboratory evaluation and follow-up; biopsy excluded inflammation and amyloidosis.	No significant differences in endoneurial microangiopathic markers between CIAP and controls without sensory polyneuropathy. Basal lamina area thickness correlated with age across the study population.	Biopsies from controls without sensory polyneuropathy were obtained in 1981–1986, compared with 2002–2009 for CIAP. Metabolic and cardiovascular risk-factor data were unavailable for these controls.
Gherardi et al., 1998 [80] Immune activation	15 CIAP and 5 chronic idiopathic ataxic neuropathy patients; three control groups of 10 each (older controls, degenerative neurological disease, CIDP).	Objective sensory neuropathy confirmed electrophysiologically; CIAP classified by sensorimotor axonal changes. Known causes, demyelination, vasculitis and mononeuritis multiplex excluded. Subacute onset was permitted.	Circulating T-cell clones in 12/15 CIAP and 4/5 ataxic neuropathies, compared with 2/10 in each control group.	Small groups; the CIAP subgroup included one subacute-onset case and five with slight asymmetry. Not a uniformly symmetric, slowly progressive cohort. Clonal expansion does not establish pathogenic autoimmunity or identify a treatment target.
Kelkar et al., 2002 [81] Inflammatory nerve pathology	11 patients selected for biopsy because of severe refractory pain. Detailed electrophysiology was available for 10; nerve conduction and needle EMG were normal in 4.	Slowly progressive distal sensory-predominant neuropathy (symptoms for 36–120 months). History, examination and laboratory studies excluded selected causes. Two-hour glucose tolerance testing and HIV serology were not performed.	Axonal loss in 9/11; multifocal loss in 4/11. Perivascular T-cell infiltrates >50 cells in 4/11 and 10–20 cells in another 4/11. No fibrinoid necrosis or transmural inflammation.	Retrospective, highly selected painful neuropathy series without a control group. Not a uniformly electrophysiologically confirmed large-fiber CIAP cohort. Immunotherapy outcomes were not reported.
Zis et al., 2021 [76] HLA associations	57 patients: 49 length-dependent neuropathy and 8 sensory ganglionopathy. HLA comparison: 106 patient alleles versus 354 alleles from 177 published English population controls.	Adults with clinical neuropathy and nerve conduction evidence of axonal damage; no recognized risk factors or other neuromuscular disorder after extensive investigation. Length-dependent neuropathy and ganglionopathy classified separately.	DQA1*05 allele frequency was higher in the pooled HLA analysis (30.2% versus 18.1%; OR 1.96, 95% CI 1.19–3.22; *p* = 0.011). HLA type was not associated with electrophysiological progression.	Small mixed-phenotype cohort; external population controls. The reported HLA odds ratio was calculated for the combined group of patients with length-dependent neuropathy and sensory ganglionopathy. HLA totals correspond to 53 patients; the difference from 57 enrolled is not explained. Independent replication in CIAP is needed; association does not establish autoimmunity.
Rouvroye et al., 2025 [82] Gluten-related antibodies	161 CIAP patients within a study of 476 patients (also small-fiber neuropathy and ataxia); 195 controls. TG6 testing used a smaller serum-available subset.	Clinical large-fiber polyneuropathy, nerve conduction studies confirming large-fiber involvement and excluding demyelination; recognized causes excluded by history, family history and laboratory investigation.	No excess gluten-related antibody positivity in CIAP. Associations at a lower antigliadin threshold concerned small-fiber neuropathy or ataxia, not CIAP.	Direct negative serological evidence for CIAP. Stored samples and incomplete TG6 availability; these antibody results cannot exclude every possible immune mechanism.
Zis et al., 2018 [83] Oxidative stress	21 CIAP and 21 age- and sex-matched gluten neuropathy patients; no healthy control group. The CIAP-labeled group included 18 patients with sensorimotor axonal polyneuropathy and 3 with sensory ganglionopathy.	Clinically and electrophysiologically confirmed large-fiber axonal neuropathy; no identified cause after extensive diagnostic workup for the CIAP group.	Protein carbonyls were higher in CIAP; sex-adjusted analyses also found higher 8-hydroxy-2′-deoxyguanosine. Biomarkers were not associated with neuropathy severity.	Small cross-sectional study from a single specialist clinic; comparison limited to gluten neuropathy, without healthy controls. Serum biomarkers do not localize oxidative damage to peripheral nerves, and the study cannot determine whether oxidative stress precedes or follows nerve injury.
Winsvold et al., 2021 [63] Common genetic susceptibility	2093 idiopathic/unspecified polyneuropathy cases and 445,256 controls across HUNT and UK Biobank.	Hospital diagnostic codes; patients with recorded diabetes were excluded. No uniform, clinically verified CIAP definition across cohorts.	Meta-analysis identified two genome-wide significant loci: chromosome 12 (lead variant within *B4GALNT3*; OR 1.19) and chromosome 1 (near *NR5A2*; OR 1.68). Of three separate HUNT discovery signals, two could not be tested in UK Biobank and one did not replicate.	Evidence from broader coded phenotypes. Association loci and candidate genes do not prove CIAP-specific causal pathways; phenotypic misclassification and replication limitations remain.
Taams et al., 2024 [84] Common genetic susceptibility	2357 chronic axonal polyneuropathy cases among 458,567 participants in Rotterdam and UK Biobank.	Rotterdam: clinical and electrophysiological assessment; UK Biobank: diagnostic codes. Acquired neuropathies of different causes were included; not restricted to CIAP.	No variant or gene association with polyneuropathy reached genome-wide significance.	Broader etiological spectrum and heterogeneous ascertainment. This is not a direct test of strictly defined CIAP; shared UK Biobank data also limit independence from earlier genetic work.
Vrancken et al., 2002 [8] Aging and CIAP severity	127 CIAP patients and 108 “successfully aging” controls.	Distal, symmetric sensory or sensorimotor neuropathy for ≥6 months; axonal electrophysiology; no cause after extensive workup or hereditary clues.	No important differences between earlier- and later-onset CIAP; superimposed aging did not importantly influence the clinical or electrophysiological phenotype.	Direct clinical evidence in CIAP, but no measurement of cellular senescence.
Taams et al., 2023 [28] Aging reference data	3780 participants without diagnosed polyneuropathy from 4179 screened in the Rotterdam Study.	Population-based neurological examination and nerve conduction studies. Definite/probable polyneuropathy excluded; possible polyneuropathy retained.	Older age was associated with lower vibration sense, fewer Achilles reflexes and lower sural sensory nerve action potential amplitudes.	Contextual evidence from people without diagnosed polyneuropathy, not a CIAP cohort. Cross-sectional age associations do not establish pathological aging or cellular senescence in CIAP.

Abbreviations: CIAP, chronic idiopathic axonal polyneuropathy; CIDP, chronic inflammatory demyelinating polyneuropathy; HMSN, hereditary motor and sensory neuropathy; EMG, electromyography; HLA, human leukocyte antigen; OR, odds ratio; CI, confidence interval; TG2/TG6, transglutaminase 2/6. Findings are observational unless stated otherwise. No row establishes disease-modifying treatment efficacy in CIAP.

### 7.1. Microvascular Hypothesis

A microvascular hypothesis has been proposed by Teunissen et al., who compared sural nerves from 18 patients with CIAP with those from four patients with diabetic neuropathy, six with hereditary motor and sensory neuropathy type II (HMSN II), and 10 autopsy controls. Basal lamina area thickness was greater in CIAP than in HMSN II, but not significantly greater than in autopsy controls; values were similar to those in diabetic neuropathy [78]. Visser et al. also suggested that a chronic inflammatory state at the level of the microvasculature, particularly involving endoneurial microvessels and endothelial cells, could lead to microvascular alterations, chronic ischemia, and, consequently, axonal injury [9]. Hube et al. compared sural nerve biopsies from 10 patients with CIAP and seven with diabetic or prediabetic polyneuropathy, without a healthy biopsy control group [31]. They found no significant differences in the number of small endoneurial vessels per fascicle, mean basal lamina thickness, or mean number of endothelial cell nuclei per capillary. These findings are compatible with overlapping microvascular changes but do not establish a shared pathogenesis. In a separate biopsy study, Samuelsson et al. found no significant differences in endoneurial microangiopathic markers between 10 patients with CIAP and 10 controls without sensory polyneuropathy [79]. These studies included only 10–18 patients with CIAP each and yielded inconsistent findings. Small samples, differences in comparator groups, and potential confounding by age and vascular comorbidity limit conclusions about a causal microvascular mechanism in CIAP. Larger studies with appropriate comparison groups are needed.

### 7.2. Neurotoxic Sphingolipids

Another hypothesis highlights the role of sphingolipids, which represent a key metabolic link between fatty acid, amino acid, and carbohydrate metabolism [85]. The de novo synthesis of sphingolipids begins with the enzyme serine palmitoyltransferase (SPT), which normally catalyzes the condensation of serine and palmitoyl-CoA to form sphinganine. However, when SPT utilizes alanine or glycine instead of serine, it produces 1-deoxy-sphingolipids (1-deoxySLs), neurotoxic lipids that lack the C1 hydroxyl group required for degradation through the canonical sphingosine-1-phosphate pathway [86]. Elevated plasma levels of 1-deoxySLs were first demonstrated in patients with hereditary sensory and autonomic neuropathy type 1 (HSAN1), carrying mutations in the *SPTLC1* gene, which encodes the first subunit of SPT [87]. Dohrn et al. subsequently evaluated the role of neurotoxic 1-deoxySLs in patients with diabetic distal symmetrical polyneuropathy and concluded that 1-deoxySLs could play a crucial role in the disease mechanism of diabetic distal symmetrical polyneuropathy [88]. Hube et al. found that plasma 1-deoxySL levels were higher in CIAP and diabetic or prediabetic polyneuropathy than in healthy controls, but did not report a comparison adjusted for metabolic syndrome or its components [31]. Because 1-deoxySLs are also elevated in metabolic syndrome, these findings do not establish an association with CIAP independent of metabolic abnormalities [31,89].

Experimental work provides a possible link between 1-deoxySL accumulation and axonal injury. In cultured cells, 1-deoxysphinganine caused mitochondrial structural and functional abnormalities; in cultured adult sensory neurons, mitochondrial changes preceded axonal degeneration [86]. These findings provide a possible cellular mechanism of toxicity but do not establish that the plasma elevations reported in CIAP cause nerve damage.

### 7.3. Inflammation and Autoimmunity

In the late 1990s, Gherardi et al. conducted a study on 20 patients with CIAP or chronic idiopathic ataxic neuropathy (CIAN), examining circulating dominant T-cell clones [80]. They concluded that both CIAN and CIAP are associated with dominant T-cell clones of unknown significance that cannot be explained solely by patient age [80]. Kelkar et al. reported findings of sural nerve biopsies from 11 patients with sensory-predominant, painful idiopathic neuropathy and found perivascular collections of T lymphocytes exceeding 50 cells in four patients (and >100 in two of them), while four additional patients showed smaller clusters of 10–20 cells. Three of the four biopsies with >50 cells also exhibited inhomogeneous myelinated fiber loss, characteristic of microangiopathic processes such as vasculitis [81]. In the aforementioned study by Hube et al., in addition to the observed similarities in sural nerve biopsies between diabetic distal symmetrical polyneuropathy and CIAP patients, an increased number of LCA (Leukocyte Common Antigen)-positive cells was found in the sural nerve biopsies of CIAP patients compared to those of patients with diabetic distal symmetrical polyneuropathy, suggesting an additional inflammatory component contributing to the pathogenesis of CIAP [31].

In aged mice, increased endoneurial macrophage numbers accompanied peripheral nerve degeneration. Depletion of macrophages through colony-stimulating factor 1 receptor inhibition reduced nerve pathology and preserved muscle strength [90]. These findings suggest that macrophages can contribute to age-related nerve injury but do not establish a corresponding mechanism or treatment target in CIAP.

The human leukocyte antigen (HLA) genes, located within the major histocompatibility complex (MHC) on chromosome 6, encode proteins essential for immune regulation. These HLA molecules function as antigen-presenting molecules, displaying self and non-self-peptides to T lymphocytes, thereby initiating and shaping the adaptive immune response [91]. The class II gene *HLA-DQA1* encodes the α-chain of the HLA-DQ heterodimer and participates in antigen presentation to CD4^+^ T cells. HLA-DQA1*05 has been associated with susceptibility to several immune-mediated diseases, particularly celiac disease, type 1 diabetes, primary Sjögren’s syndrome, systemic lupus erythematosus, inflammatory myopathies, membranous nephropathy, and several others [92,93,94,95,96,97,98]. How specific HLA alleles contribute to disease pathogenesis remains unknown. Further exploring the autoimmune hypothesis of CIAP, Zis et al. investigated HLA genetic susceptibility in 57 patients with idiopathic axonal neuropathy, comprising 49 with length-dependent neuropathy and eight with sensory ganglionopathy. In the pooled HLA analysis, the HLA-DQA1*05 allele frequency was higher in patients than in published population controls (30.2% versus 18.1%; odds ratio 1.96, 95% confidence interval 1.19–3.22; *p* = 0.011). To our knowledge, this association has not been independently replicated in a clinically defined CIAP cohort. It does not establish an autoimmune mechanism in CIAP [76].

Anti-gliadin antibodies have previously been associated with idiopathic neurological disorders such as ataxia, neuropathy, myelopathy, and myopathy [99]. Based on this hypothesis, Lock et al. conducted a study including seven patients with hereditary ataxia, 20 with idiopathic ataxia, and 32 with idiopathic peripheral neuropathy. Although their findings did not demonstrate a frequent association between coeliac disease and either idiopathic ataxia or idiopathic peripheral neuropathy, and suggested that anti-gliadin antibody positivity in these patients may be non-specific, the limited sample size restricts the strength of these conclusions and does not exclude a potential association in larger or more selected populations [100]. More recently, Rouvroye et al. analyzed the prevalence of gluten-related antibodies in a large cohort of patients with idiopathic cerebellar ataxia, idiopathic small-fiber neuropathy, and CIAP in comparison to healthy controls [82]. Their main finding was that there were no differences between disease groups and controls for anti-gliadin antibodies when applying the traditional cut-off value (10 U/mL), nor for anti-tissue transglutaminase 6 or anti-tissue transglutaminase 2 antibodies. Only when using a lower cut-off value (3 U/mL) was the prevalence of these antibodies higher in patients with idiopathic cerebellar ataxia and idiopathic small-fiber neuropathy. In CIAP patients, no difference in antibody prevalence was observed, even when applying the lower threshold [82].

### 7.4. Genetic Susceptibility and Diagnostic Reclassification

Genetic testing can reveal an inherited neuropathy in patients initially labeled as having CIAP, prompting diagnostic reclassification. This differs from the proposed biological stratification of patients whose neuropathy remains idiopathic. Susceptibility associations alone, however, do not establish a genetic diagnosis.

Winsvold et al. conducted the first genome-wide association study (GWAS) of idiopathic polyneuropathy using data from two large population-based health studies. These were the Norwegian Trøndelag Health Study (1147 cases and 62,204 controls) and UK Biobank (946 cases and 383,052 controls). The combined meta-analysis identified two independent genome-wide significant susceptibility loci: on chromosome 12, with the lead variant within *B4GALNT3*, and on chromosome 1, near *NR5A2* [63]. Other candidate genes discussed at the chromosome 12 locus included *NINJ2* and *KDM5A*. At the chromosome 1 locus, expression quantitative trait locus (eQTL) mapping linked the locus to *INAVA* expression in leukocytes. These analyses did not establish which genes mediate the associations. *NINJ2*, which encodes ninjurin-2 (nerve injury-induced protein 2), plays a role in nerve regeneration following injury. *KDM5A* encodes lysine demethylase 5A, an epigenetic regulator implicated in neurodevelopmental disorders. *NR5A2*, encoding liver receptor homolog-1 (LRH-1), is involved in bile acid metabolism, glucose homeostasis, and carcinogenesis. Finally, *INAVA*, which encodes the innate immunity activator protein, has been associated with inflammatory bowel disease and appears to play a critical role in macrophage regulation, highlighting a possible autoimmune component in the pathogenesis of idiopathic neuropathies [63]. So far, no study has validated these results. Taams et al. failed to reproduce these findings even though both studies used the data from the UK Biobank [84]. Neither study applied strict diagnostic criteria for CIAP. Taams et al. included participants with chronic axonal polyneuropathy irrespective of etiology. Winsvold et al. defined cases using ICD-10/9 codes for polyneuropathy recorded in medical records. These comprised idiopathic progressive neuropathy (ICD-10 G60.3, ICD-9 356.4), other specified idiopathic peripheral neuropathy (ICD-9 356.8), unspecified hereditary and idiopathic neuropathy (ICD-10 G60.9), and unspecified polyneuropathy (ICD-10 G62.9, ICD-9 356.9). While this approach enabled large-scale analyses, it reduced the certainty that the included cases represented a truly pure CIAP cohort.

Rare variants in *MME*, encoding the membrane-bound zinc metalloprotease neprilysin, have been implicated in late-onset axonal polyneuropathies [101,102,103,104,105,106]. Reported neuropathy-associated variants are predominantly coding missense or loss-of-function variants, although splice-site variants have also been described. Auer-Grumbach et al. identified heterozygous rare loss-of-function and missense *MME* variants in patients with late-onset Charcot-Marie-Tooth type 2, and the variants segregated in an autosomal-dominant pattern with age-related incomplete penetrance. In this study, *MME* variants were associated with reduced neprilysin tissue availability and impaired enzymatic activity. Senderek et al. later performed whole-exome sequencing (WES) and *MME* single-gene sequencing in 230 patients with unexplained axonal neuropathies and disease onset at or after 35 years [101]. In the WES cohort of 126 probands, the overall detection rate for presumed disease-causing variants in genes associated with hereditary neuropathies or other conditions featuring peripheral neuropathy was 18.3% (n = 23). The most frequently implicated gene was *MME*: five patients carried single heterozygous loss-of-function variants (nonsense, frameshift, or splice-site), and three patients harbored rare biallelic variants. Other affected genes included *LRSAM1* (n = 3), *MPZ* (n = 2), and *TTR* (n = 2). The authors subsequently conducted targeted sequencing of *MME* in an independent cohort of 104 individuals, identifying private or rare *MME* variants in 14 cases. The relevance of *MME* to late-onset neuropathies was further supported by an increased frequency of *MME* variants in patients compared with population reference data, the identification of additional late-onset neuropathy cases with *MME* variants in WES repositories, and reduced neprilysin levels in patients’ blood samples. However, Senderek et al. emphasized that their findings may not be readily generalizable to patient cohorts with different genealogical backgrounds or clinical phenotypes, such as less progressive or purely sensory neuropathies, which may limit comparability with CIAP. A detailed discussion of the study and its implications has been provided by Shy [107]. *MME* variants have also been associated with spinocerebellar ataxia with neuropathy (SCA43), distal hereditary motor neuropathy, and membranous glomerulonephritis [108,109,110].

Biallelic pentanucleotide repeat expansions in replication factor C subunit 1 (*RFC1*) are a known cause of Cerebellar Ataxia, Neuropathy, and Vestibular Areflexia Syndrome (CANVAS) [111,112]. However, pathogenic biallelic *RFC1* repeat expansions have been identified as a cause of sensory neuropathy previously considered idiopathic. Currò et al. retrospectively analyzed 225 patients initially diagnosed with CIAP (125 with sensory neuropathy and 100 with sensorimotor neuropathy) who underwent *RFC1* testing [17]. Biallelic *RFC1* expansions were identified in 34% of patients with sensory neuropathy and in none of those with sensorimotor neuropathy. Among *RFC1*-positive patients, 42% presented with isolated sensory neuropathy or sensory neuropathy accompanied by chronic cough, whereas vestibular and/or cerebellar involvement, often subclinical, was observed in 58%. Similarly, Tagliapietra et al. screened 234 probands initially diagnosed with CIAP for pathogenic biallelic *RFC1* AAGGG repeat expansions and found that these expansions were common in patients with pure sensory neuropathy (53%) and less frequent in cases with predominantly sensory (18%) or sensorimotor (2%) neuropathy [113]. To date, monoallelic RFC1 AAGGG repeat expansions have not been associated with an increased risk of developing polyneuropathy. Functional data suggest that large intronic *RFC1 AAGGG* expansions cause disease through a toxic repeat gain-of-function mechanism rather than *RFC1* loss of function, as *RFC1* mRNA and protein levels appear largely preserved in patient tissues and induced pluripotent stem cell-derived neurons [114]. Overall, in patients labeled CIAP who have pure sensory neuropathy, sensory ataxia, chronic cough, vestibular symptoms, cerebellar signs, or non-length-dependent sensory involvement, *RFC1* testing should be considered before accepting the diagnosis of CIAP.

### 7.5. Oxidative Stress

Oxidative stress has been found to play a substantial role in the development and progression of diabetic distal symmetrical polyneuropathy [115]. Following this premise, Zis et al. analyzed serum samples from 21 patients with CIAP and compared them with 21 age- and sex-matched patients with gluten neuropathy. Titers of two markers of oxidative stress (8-hydroxy-2′-deoxyguanosine and protein carbonyl) were measured [83]. No correlations were observed between these markers and age, neuropathy type, or disease severity in either group. In univariate analyses, patients with CIAP exhibited significantly higher protein carbonyl levels compared with patients with gluten neuropathy. After adjustment for sex, CIAP patients showed significantly higher titers of both 8-hydroxy-2′-deoxyguanosine and protein carbonyl. On this basis, the authors suggested that oxidative stress may be increased in CIAP and could contribute to its pathogenesis [83]. However, given that gluten neuropathy is itself an inflammatory condition [116], comparison with a healthy control group may have provided a more appropriate reference for assessing disease-specific increases in oxidative stress.

### 7.6. Age-Related Peripheral Nerve Changes

Aging and cellular senescence are related but distinct processes. Cellular senescence involves a stress-associated state with altered metabolism and secretory activity and, in proliferating cells, stable cell-cycle arrest [117]. The clinical studies discussed below document age-related peripheral nerve changes but do not directly assess cellular senescence. We therefore refer to age-related axonal vulnerability as a proposed susceptibility factor in CIAP. Many older individuals without diagnosed polyneuropathy show neuropathy signs on neurological examination, including absent distal vibration sense, absent ankle reflexes, and reduced sural SNAP amplitudes [28,118,119]. Taams et al. conducted a large prospective population-based study involving 4179 participants, of whom 3780 did not fulfill the criteria for polyneuropathy, and reported that the prevalence of abnormalities at the neurological examination increased with age, most notably regarding impaired vibration sense at the hallux and decreased Achilles tendon reflexes. In contrast, superficial pain sensation and patellar tendon reflexes remained relatively stable over time, suggesting that these features may be more informative in older age. Regarding NCS, sural SNAP declined progressively with increasing age, with a faster decline observed in advanced age. Furthermore, nearly one third of participants older than 80 years had nonrecordable sural SNAP amplitudes [28]. Such age-related changes should be considered in the evaluation of older patients with suspected chronic axonal polyneuropathy.

To investigate whether CIAP is influenced by these overlapping effects of aging, Vrancken et al. studied 127 patients with CIAP and 108 age-matched healthy controls [8]. They assessed the relationship between disease severity, duration, and age-related changes, and concluded that neither aging of the peripheral nervous system nor disease duration significantly impacts the severity or progression of CIAP [8]. Vrancken et al. also discussed that symptoms such as mild pure sensory or pure motor manifestations, slight and purely sensory signs limited to the feet (including absent vibration sense at the big toe), or absent ankle reflexes may be regarded as normal aging-related findings in elderly individuals over 65 years [8].

The available pathological and biomarker findings support further investigation of vascular, metabolic, inflammatory and oxidative pathways but do not establish their causal role in CIAP or define validated mechanistic subgroups. Age-related peripheral nerve changes are well documented, but their contribution to CIAP remains uncertain. The proposed interaction between age-related axonal vulnerability and additional “second hits” therefore remains a working hypothesis.

Neurofilament light chain (NfL) is a candidate blood biomarker of axonal injury. In a cohort of idiopathic peripheral neuropathy that included both large- and small-fiber phenotypes, elevated plasma NfL was associated with greater clinical severity and lower peroneal motor and sural sensory response amplitudes, but most participants had age-adjusted values within the normal range [120]. Measurements were cross-sectional, and these findings do not establish NfL as a marker of progression or possible treatment response in CIAP.

Future studies could examine whether metabolic abnormalities and circulating biomarkers are associated with the clinical phenotype or progression of CIAP. In cohorts using uniform diagnostic criteria, candidate measures include abdominal obesity and hypertension, plasma 1-deoxysphinganine and 1-deoxysphingosine, and serum protein carbonyls and 8-hydroxy-2′-deoxyguanosine. Baseline measurements could be related to motor involvement, neuropathic pain, and subsequent changes in sensory and motor nerve response amplitudes and disability. Analyses should account for age, sex and disease duration and assess whether biomarker associations persist after adjustment for metabolic syndrome components. Biomarker concentrations could initially be analyzed as continuous variables, since thresholds for defining CIAP subgroups have not been validated. Associations with subsequent progression would require confirmation in independent cohorts. Table 3 summarizes the candidate features and potential therapeutic relevance.

## 8. Disease Burden and Quality of Life

Although CIAP usually progresses slowly, relatively few studies have examined its impact on quality of life. It is well established that patients with chronic polyneuropathy experience difficulties performing basic activities of daily living, particularly those requiring mobility [121]. In a study conducted 25 years ago, Teunissen et al. showed that patients with chronic axonal polyneuropathy (66 with CIAP and 39 with hereditary motor and sensory neuropathy type 2) scored worse than the reference population in seven of eight domains of the RAND 36-item Health Survey (RAND-36), with the greatest differences observed in the “social functioning” domain [122]. The two groups differed only in the “vitality” domain, where patients with hereditary motor and sensory neuropathy type 2 scored worse than CIAP patients. These findings suggest that the considerable negative impact of CIAP on quality of life (QoL) may be underestimated due to the comparatively benign character of the disease [122]. Later studies comparing QoL in CIAP patients with that of the general population also reported reduced QoL in CIAP [123]. The presence of pain was associated with a worse QoL in the energy/fatigue, emotional well-being, and general health perception domains in a study that included 55 CIAP patients [124]. There are no studies examining the socioeconomic burden of CIAP.

## 9. Treatment

No established disease-modifying therapy is available for CIAP. Management therefore remains primarily symptomatic.

### 9.1. Evidence from CIAP Cohorts

The 2017 Cochrane review assessed 42 studies for possible inclusion but found none that met its eligibility criteria [10]. A published protocol describes the EPHENE trial, designed to evaluate the efficacy and safety of topical phenytoin cream in patients with painful CIAP [125]. In a small, uncontrolled study of caudal epidural pulsed radiofrequency, 10 of 20 patients with CIAP and refractory neuropathic pain reported at least 50% pain reduction after three months [126]. These interventions target pain relief rather than modification of the underlying neuropathy.

### 9.2. Symptomatic Management and Evidence from Related Neuropathies

Treatment of neuropathic pain in CIAP draws largely on general neuropathic pain recommendations and evidence from related neuropathy populations [127]. The open-label PAIN-CONTRoLS trial compared nortriptyline, duloxetine, pregabalin, and mexiletine in 402 participants aged ≥30 years with physician-diagnosed cryptogenic sensory polyneuropathy and pain scores ≥4/10. At 12 weeks, ≥50% pain reduction while remaining on the assigned treatment was achieved by 34/134 (25.4%), 29/126 (23.0%), 11/73 (15.1%), and 14/69 (20.3%), respectively. Although no single medication demonstrated clear superiority, nortriptyline and duloxetine performed better when efficacy and treatment discontinuation were considered together. Known causes of neuropathy, including diabetes, were excluded, but investigations for underlying causes were not repeated within the trial. The trial therefore provides evidence in clinically diagnosed cryptogenic sensory polyneuropathy, without establishing that all participants met the CIAP definition used in this review [128]. Similarly, sustained pain relief with topical phenytoin was reported in a retrospective study of patients with painful diabetic neuropathy selected for a positive initial response test [129]; its applicability to CIAP remains uncertain.

Control of cardiovascular and metabolic risk factors should also be considered as part of general care [9]. Elafros et al. highlighted dietary and surgical weight loss and exercise as promising interventions for obesity-related neuropathy [130], but whether these approaches alter the course of CIAP remains unproven. Bariatric surgery may cause nutritional deficiencies, particularly after malabsorptive procedures, requiring careful monitoring and targeted management [131].

### 9.3. Experimental and Hypothetical Disease-Modifying Approaches

Immunomodulatory approaches, including intravenous immunoglobulin, plasma exchange, corticosteroids, and rituximab, have been attempted in previous studies; however, these investigations involved heterogeneous patient populations and very small cohorts based mostly on case reports [132,133,134,135]. These reports provide insufficient evidence of disease-modifying efficacy in CIAP. Warendorf et al. also discussed riluzole, tacrolimus derivatives without immunosuppressive properties, Rho kinase inhibitors, neuroactive steroids, and nitric oxide synthase inhibitors as potential avenues for future research, drawing on experimental findings and evidence from other neurological disorders [10]. Their efficacy in CIAP remains unproven.

## 10. Conclusion and Future Directions

The first issue that requires clarification is the definition of CIAP itself. An expert consensus on the terminology to be used in future studies is needed. In this context, ACTTION terminology (iDSP and its subtypes) may be preferable for characterizing sensory phenotypes, with iMFN used when the corresponding criteria are met. Any study-specific exception permitting motor involvement is possible according to the ACTTION criteria but should be stated explicitly in the eligibility criteria. Where skin biopsy is unavailable, small-fiber symptoms and signs should still be documented clinically, and the diagnostic tests supporting phenotype classification should be reported. CIAP may nevertheless remain useful as an established clinical designation for chronic idiopathic axonal sensory or sensorimotor polyneuropathy, provided that the diagnostic criteria and phenotype are clearly specified.

Furthermore, current diagnostic methods used to assess peripheral nervous system function, such as NCS, may not be entirely accurate. In the authors’ clinical experience, patients with chronic polyneuropathies often present with very cold distal extremities. As previously mentioned, applying temperature correction factors instead of adequately warming the extremities may not be appropriate in axonal polyneuropathies. Nonetheless, proper warming is time-consuming and is likely neglected in everyday clinical practice. Beyond NCS, there is a clear need for additional biomarkers in axonal polyneuropathies to allow for a more accurate and objective assessment of patients. This raises the question of whether patients who present with sensory symptoms but have no weakness, have normal NCS, and have normal skin biopsies should be classified as having “no polyneuropathy”, or whether our current diagnostic tests simply lack sufficient sensitivity. Indeed, the present diagnostic framework may incorrectly categorize such patients as unaffected, even though subclinical small-fiber or early axonal large-fiber dysfunction may be present. This underscores the need for more sensitive biomarkers and improved phenotyping strategies.

CIAP most likely represents a heterogeneous group of disorders that manifest with very similar clinical features. Among patients whose neuropathy remains idiopathic, unraveling this heterogeneity and clustering patients according to shared pathomechanisms using a multi-omics approach could provide a basis for identifying subgroups who may respond to specific treatments in future clinical trials. For example, identifying a subgroup with reproducible evidence of immune-mediated nerve injury could provide a rationale for controlled trials of immunomodulatory therapy.

Future research should focus on four priorities: harmonizing diagnostic criteria and documenting sensory and motor phenotypes; prospectively assessing whether metabolic abnormalities and circulating biomarkers are associated with changes in nerve conduction and disability; confirming these associations in independent cohorts after accounting for age, sex, disease duration and metabolic risk factors; and, where reproducible evidence supports a proposed subgroup, testing targeted interventions in controlled trials.

## Figures and Tables

**Table 1:** Typical clinical and electrophysiological patterns of pure SFN, CIAP and SSN.

Feature	Pure SFN	CIAP	SSN
Primary target	Small fibers (Aδ/C)	Distal axons, predominantly large fibers; small fibers may also be involved	Dorsal root ganglion
Typical onset/course	Slowly progressive, insidious	Slowly progressive, insidious	Subacute (weeks-months) to rapidly progressive
Symptom distribution	Usually length-dependent	Length-dependent	Non-length-dependent
Symmetry	Symmetric	Symmetric	Often asymmetric
Positive sensory symptoms	Prominent (burning pain, dysesthesia)	Mild-moderate	Variable
Negative sensory symptoms	Mild	Prominent (numbness)	Severe
Sensory ataxia	Absent	Absent or mild	Frequent
Autonomic involvement	Frequent	Reported; frequency uncertain	Possible
Motor weakness	Absent	May be present; usually mild and distal	Absent
Deep tendon reflexes	Preserved	Reduced distally	Early loss
Electrophysiology			
SNAP amplitude	Normal	Reduced	Severely reduced or absent
SNAP asymmetry	No	Usually no	Yes
Upper vs. lower limb SNAP involvement	No involvement	lower > upper	upper ≥ lower
Motor NCS	Normal	Normal or mildly abnormal	Normal
EMG	Normal	May show chronic neurogenic changes	Normal

SFN—small-fiber neuropathy; CIAP—chronic idiopathic axonal polyneuropathy; SSN—subacute sensory neuronopathy; SNAP—sensory nerve action potential; NCS—nerve conduction studies; EMG—electromyography; The table summarizes typical patterns rather than formal diagnostic criteria. Sources: pure SFN [2,11]; CIAP [20,21,22]; SSN [19].

**Table 3:** Candidate features for mechanistic stratification of CIAP and potential therapeutic relevance.

Proposed Mechanism or Genetic Category	Candidate Features for Assessment	Therapeutic Relevance
Metabolic and microvascular mechanisms [9,31,78,79]	Metabolic syndrome components, particularly hypertension and abdominal obesity; endoneurial vessel basal lamina thickening reported in some biopsy comparisons.	Management of cardiometabolic risk factors remains part of general care; whether it also influences CIAP progression is unclear.
1-Deoxysphingolipid abnormalities [31]	Plasma 1-deoxysphinganine and 1-deoxysphingosine concentrations, assessed together with metabolic syndrome components.	These findings suggest a possible rationale for studying interventions that lower 1-deoxysphingolipids, although benefit in CIAP remains to be demonstrated.
Inflammatory or immune mechanisms [76,80,81]	Circulating T-cell clonality; HLA-DQA1*05 carriage; perivascular inflammatory infiltrates in available nerve biopsies.	Whether these findings can help identify patients who benefit from immunotherapy remains to be determined.
Oxidative stress [83]	Serum protein carbonyls and 8-hydroxy-2′-deoxyguanosine.	These observations may support further investigation of oxidative stress; their value for guiding antioxidant treatment remains uncertain.
Common genetic susceptibility [63,84]	Genotyping of susceptibility loci reported in idiopathic or chronic axonal polyneuropathy cohorts. The associations reported by Winsvold et al. were not confirmed by Taams et al. after correction for multiple testing.	Their relevance to treatment selection in CIAP is not yet established.
Age-related axonal vulnerability [8,28]	Age at onset and examination; distal vibration sense, ankle reflexes and sural sensory nerve action potential amplitudes interpreted using age-appropriate reference values.	Whether age-related axonal vulnerability can be modified therapeutically in CIAP remains uncertain.
Confirmed inherited neuropathy: diagnostic reclassification [15,17,101,113]	Clinically concordant disease-causing findings, such as pathogenic biallelic *RFC1* expansions or an established *MME*- or *TTR*-related diagnosis.	Can inform genetic counseling and management according to the established diagnosis.

The first six rows describe candidate mechanisms or susceptibility factors; the final row concerns an alternative diagnosis identified during the evaluation of presumed CIAP. CIAP-specific biomarker thresholds for mechanistic classification remain to be validated. Biopsy findings are included as evidence from selected studies, not as a proposed requirement for subgroup assignment. Study findings and limitations are summarized in Table 2. CIAP, chronic idiopathic axonal polyneuropathy; HLA, human leukocyte antigen; RFC1, replication factor C subunit 1; MME, membrane metalloendopeptidase; TTR, transthyretin.

## Data Availability

No new data were created or analyzed in this study. Data sharing is not applicable to this article.
